# Strong Country Level Correlation between Syphilis and HSV-2 Prevalence

**DOI:** 10.1155/2016/5959032

**Published:** 2016-03-16

**Authors:** Chris Richard Kenyon, Achilleas Tsoumanis, Kara Osbak

**Affiliations:** ^1^HIV/STI Unit, Institute of Tropical Medicine, Nationalestraat 155, Antwerp, Belgium; ^2^Division of Infectious Diseases and HIV Medicine, University of Cape Town, Anzio Road, Observatory, Cape Town 7700, South Africa; ^3^Clinical Trials Unit, Institute of Tropical Medicine, Antwerp, Belgium

## Abstract

*Background.* Syphilis is curable but Herpes Simplex Virus-2 (HSV-2) is not. As a result, the prevalence of syphilis but not HSV-2 may be influenced by the efficacy of national STI screening and treatment capacity. If the prevalence of syphilis and HSV-2 is found to be correlated, then this makes it more likely that something other than differential STI treatment is responsible for variations in the prevalence of both HSV-2 and syphilis.* Methods.* Simple linear regression was used to evaluate the relationship between national antenatal syphilis prevalence and HSV-2 prevalence in women in two time periods: 1990–1999 and 2008. Adjustments were performed for the laboratory syphilis testing algorithm used and the prevalence of circumcision.* Results.* The prevalence of syphilis was positively correlated with that of HSV-2 for both time periods (adjusted correlations, 20–24-year-olds: 1990–99: *R* ^2^ = 0.54, *P* < 0.001; 2008: *R* ^2^ = 0.41, *P* < 0.001 and 40–44-year-olds: 1990–99: *R* ^2^ = 0.42, *P* < 0.001; 2008: *R* ^2^ = 0.49, *P* < 0.001).* Conclusion.* The prevalence of syphilis and HSV-2 is positively correlated. This could be due to a common set of risk factors underpinning both STIs.

## 1. Introduction

**Figure 1:**
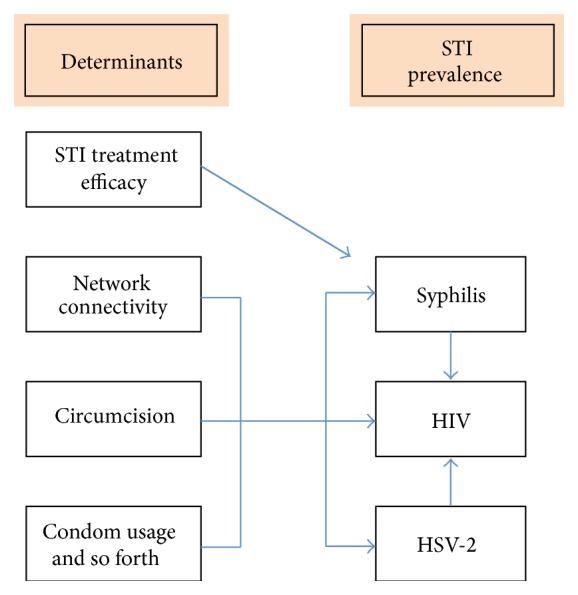
Conceptual framework illustrating the relationship between syphilis, HIV, and HSV-2 prevalence and four underlying risk factors. Syphilis prevalence is directly and HIV prevalence is indirectly influenced by STI treatment efficacy. HSV-2 is not influenced by STI treatment efficacy.

Is there a correlation between the national prevalence of syphilis and Herpes Simplex Virus-2 (HSV-2)? The answer to this question may provide useful information on the determinants of differences in STI prevalence. It has long been argued that STI screening and treatment efficacy (STI-STE) was instrumental in the large reductions in syphilis prevalence seen in the mid-20th century in many countries [1]. Differential STI-STE was also thought to have played a large role in explaining why some countries experienced greater declines in the prevalence of syphilis and other treatable STIs than others [1, 2]. STI-STE should not however influence the prevalence of incurable STIs such as HSV-2 [3]. Whilst studies have demonstrated a reduction in transmission of HSV-2 in discordant couples taking antiherpes virus therapy [4] no population in the world has taken sufficient antiherpes therapy to make this a plausible explanation for the large international differences in HSV-2 prevalence [5]. The prevalence of HSV-2 can therefore be thought of as a measure of the prevalence of non-STI-STE related risk factors in a population [6]. It is more useful in this regard than HIV as, unlike HIV, HSV-2's transmission/acquisition has not been shown to be enhanced by the presence of other STIs such as syphilis [7, 8]. The relationship between HIV, HSV-2, syphilis, and their risk factors is illustrated in Figure 1. As shown, the prevalence of HIV (but not HSV-2) could be indirectly influenced by STI-STE via the intervening variable of syphilis prevalence. Thus assessing the correlation between syphilis and HSV-2 could shed light on the determinants of variations in STI prevalence. If we find no correlation then it is more likely that nonshared risk factors (such as STI-STE) are responsible. If we find a correlation then this makes it more probable that shared risk factors are responsible and that STI-STE is less likely to be a major player in this regard.

In this paper we assess if the prevalence of syphilis is correlated with HSV-2 prevalence at a country level in two time periods: 1990–99 and 2008. We include two time periods as there was a dramatic decline in syphilis prevalence at the end of the 20th century in the previously hyperendemic countries in sub-Saharan Africa [9, 10]. This decline was likely due to a combination of factors but one of these was the effect of AIDS mortality on the structure of sexual networks [9, 11, 12]. If AIDS mortality disrupted sexual network connectivity sufficiently to reduce syphilis prevalence then this could diminish or eliminate a relationship between HSV-2 and syphilis prevalence. AIDS mortality would have had less impact on the prevalence of syphilis in 1990–1999 than later periods, as this period was early in the AIDS epidemic for most countries.

## 2. Methods

### 2.1. Data

#### 2.1.1. ASP 1990–99

Antenatal syphilis data for 1990 to 1999 were taken from an Institute for Health Metrics and Evaluation (IHME) database on the prevalence of syphilis. IHME compiled all available syphilis seroprevalence data in low risk populations, which was predominantly comprised of pregnant women receiving antenatal care. Data sources included UNAIDS epidemiologic fact sheets, UNGASS country progress reports, country specific surveillance systems where available, WHO reports on syphilis epidemiology, and unpublished data from correspondence with Global Burden of Disease 2010 collaborators. These were supplemented by a systematic literature review of syphilis seroprevalence (most recent PubMed search was October 2011). To be included, each study needed to provide data on the prevalence of syphilis in populations considered representative of the general population and have a sample size of 100 [13–16].

We extracted the studies conducted on antenatal populations to construct the national prevalence of antenatal syphilis (*syphilis prevalence*) variable as follows: the median prevalence of syphilis (expressed as a percentage) in all studies performed between the years 1990–1999 in antenatal women in a particular country.

For the* adjusted syphilis prevalence* we corrected each syphilis prevalence estimate according to the syphilis testing algorithm used since different types of testing algorithms influence the estimates of prevalence. Studies that use both a treponemal and a nontreponemal test to diagnose infection are regarded as offering the most accurate measure of active syphilis infection [17]. We applied correction factors to different syphilis testing algorithms used based on a systematic review and meta-analysis that estimated the proportion of pregnancies with “probable active syphilis” according to the testing methodology used in the study [18].

#### 2.1.2. *2008* ASP

Researchers from the World Health Organization (WHO) published estimates of antenatal syphilis prevalence for 2008 [19]. These were based on a variety of sources and were published as unadjusted and adjusted (for testing methodology) estimates.

### 2.2. HSV-2 Prevalence

National HSV-2 prevalence estimates for 1990–99 and 2000–13 were obtained from three systematic reviews of global HSV-2 incidence and prevalence conducted in 2002, 2005, and 2015 [5, 19, 20]. All three reviews restricted the data to that from peer-reviewed articles that used a type-specific serological methodology for the detection of HSV-2. Only articles that had sample sizes greater than 20 per age group, that detailed age-specific rates, and that were sampled in the period from 1990 to 1999 were included.

We used two indicators of HSV-2 prevalence: HSV-2 prevalence in 20–24-year-old and 40–44-year-old women (expressed as a percentage of all women in the respective age category who tested seropositive for HSV-2). The rationale for choosing these indicators is that HSV-2 seroprevalence increases monotonically with age but at different rates depending on both gender and the population concerned. The rate of increase is greater in women. The data for age groups above 45 years are, however, poorly populated. HSV-2 prevalence in 40–44-year-old women is thus an indicator for which the available data are relatively complete and an indicator that will most likely exhibit differences in HSV-2 prevalence between populations. 20–24-year-old prevalence has been proposed as composite marker of risk factors of young populations [6, 19]. These risk factors would include number of partners, partner concurrency, and age mixing [6]. One study found that no country where HSV-2 prevalence in this age group was below 20% went on to have a generalized HIV epidemic [6].

### 2.3. Circumcision

We controlled for the national circumcision prevalence in our adjusted analyses, since circumcision has been shown to reduce the acquisition of both these STIs in men, which in turn likely results in a lower prevalence of these infections in populations where circumcision is more uniform [21–23].

The national prevalence rates of male circumcision as of December 2006 were taken from World Health Organization and Joint United Nations Programme on HIV/AIDS publication. These estimates were based on Demographic and Health Survey data or from other published sources [24]. Countries were classified as having circumcision prevalence rates <20%, 20–80%, or >80%.

### 2.4. Statistical Analysis

Simple linear regression was used to evaluate the relationship between syphilis prevalence and HSV-2 prevalence: unadjusted, adjusted for laboratory testing used, and both of these adjusted for circumcision prevalence. We also mapped ASP, HSV-2, and peak HIV prevalence by country. Peak HIV prevalence was defined as the peak prevalence that HIV reached in each country up until 2008. Peak HIV prevalence figures were taken from a publication that calculated these figures based on UNAIDS prevalence estimates [25]. The peak HIV prevalence map was included for the purpose of visual comparison only. All analyses were performed in R v. 3.2.0 (R Foundation, Austria).

## 3. Results

**Figure 2:**
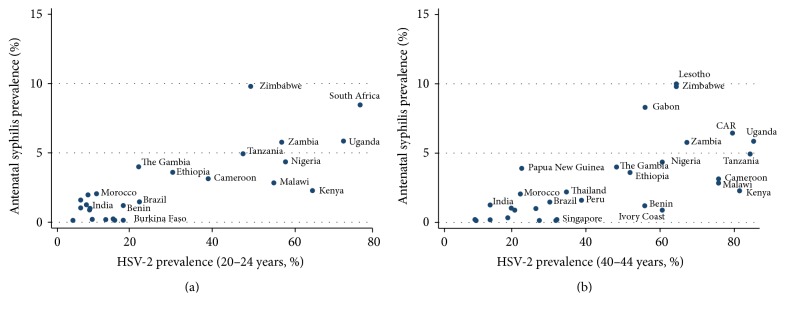
Association between unadjusted antenatal syphilis prevalence and national HSV-2 prevalence and 20–24-year-old women (a) and 40–44-year-old women (b), all data from 1990–1999.

In both time periods, we found a positive association between syphilis prevalence and 20–24-year-olds HSV-2 prevalence (1990–99: *R* ^2^ = 0.59, *P* < 0.001, *n* = 32; 2008: *R* ^2^ = 0.41, *P* < 0.001, *n* = 31; Figure 2) and 40–44-year-olds HSV-2 prevalence (1990–99: *R* ^2^ = 0.41, *P* < 0.001, *n* = 26; 2008: *R* ^2^ = 0.49, *P* < 0.001, *n* = 36). The association was somewhat weakened but remained significantly positive when we adjusted for the type of syphilis testing used (20–24-year-olds: 1990–99: *R* ^2^ = 0.54, *P* < 0.001; 2008: *R* ^2^ = 0.41, *P* < 0.001 and 40–44-year-olds: 1990–99: *R* ^2^ = 0.42, *P* < 0.001; 2008: *R* ^2^ = 0.49, *P* < 0.001). The inclusion of the circumcision prevalence variable did not attenuate the association for the adjusted and unadjusted estimates (*P* remained <0.001 for both age groups in both time periods).

**Figure 3:**
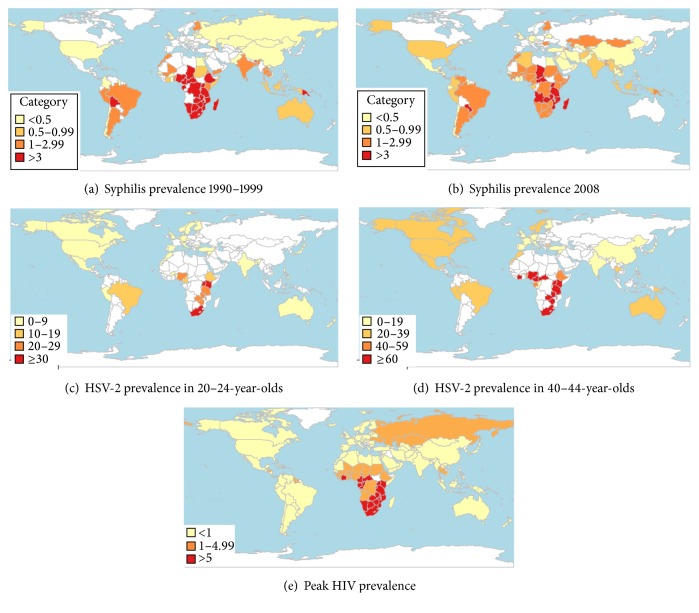
The antenatal prevalence of syphilis by country in 1990–1999 (a) and 2008 (b), unadjusted for testing strategy, the 1990–1999 prevalence of HSV-2 by country in 20–24-year-old women (c) and 40–44-year-old women (d), and the peak prevalence of HIV by country (e).

The HSV-2, syphilis, and peak HIV prevalence maps showed a considerable overlap in the countries and world regions most heavily affected by the three STIs (Figure 3). All the countries in the highest prevalence category for HSV-2 in the 1990s for both 20–24-year-olds and 40–44-year-olds were in sub-Saharan Africa. The same was true for syphilis in the 1990s (with two exceptions) and in 2008 (with one exception). All the countries in the highest peak HIV prevalence category were also in sub-Saharan Africa.

## 4. Discussion

Previous studies have found national peak HIV prevalence to be associated with both HSV-2 [6] and antenatal syphilis prevalence [26]. Because both syphilis and HSV-2 can enhance HIV transmission/acquisition, these associations could be explained on the basis of this biological effect [23, 27]. There is no evidence that we can find for either HSV-2 or syphilis enhancing the spread of the other [27]. This creates the rationale for our analysis. We find evidence of a positive association between syphilis prevalence and HSV-2 prevalence at a country level in both time periods. A similar positive association has previously been found at the level of ethnic groups within specific countries [28]. As argued above, this is most parsimoniously explained by both HSV-2 and syphilis prevalence being driven by a common risk factor(s) that is not STI-STE.

There are however a number of other possible interpretations. Our results are based on a relatively low number of data points and may represent chance findings. There are significant limitations in both the syphilis and the HSV-2 prevalence estimates. Both are taken from reviews that assembled the available data but there were a number of limitations in the data. A large number of countries had no data available. Of the countries with data, prevalence estimates were sometimes based on relatively small or somewhat selected samples that may not be representative of the general population. We only controlled for the effect of circumcision rates. The effect of each of these data and methodological limitations should however dilute the strength of an association between syphilis and HSV-2 prevalence. This may explain why the strength of the correlation we found would only explain approximately 40 to 60% of the variation in HSV-2 and syphilis prevalence. Other factors that could explain the residual difference are STI-STE and differences between HSV-2 and syphilis in terms of the biology of infection and transmissibility.

Whilst STI-STE is a crucial component of STI control, there is increasing evidence that this may be insufficient to reduce high STI rates in affected populations. At least 8 randomized controlled trials have now been published involving interventions trying to reduce the incidence of HIV by treating STIs [3]. Only one has shown evidence of an effect [29]. Our study adds to the evidence base that suggests that high STI prevalence is determined not only by poor STI-STE and lower circumcision prevalence but also by other factors, which could include low condom usage and the connectivity of local sex networks [30].

Other studies have provided evidence that one of the determinants of sexual network connectivity, partner concurrency, is strongly associated with both HSV-2 and syphilis at individual [31, 32] and population levels [6, 33]. These findings suggest that one way to explain the global variations in STIs over the last 25 years is that populations with more connected sexual networks had higher prevalence of HSV-2 and syphilis prior to the HIV epidemic and went on having high peak HIV prevalence [34]. The patterning of HIV, HSV-2, and syphilis presented here is compatible with this thesis. If a common set of risk factors were responsible for some populations having higher rates of HIV, HSV-2, and syphilis then this would have important consequences for STI prevention activities. One future study design that could shed further light on these issues would be a study that follows up a cohort of teenagers from a town in a country such as South Africa that has communities with low, medium, and very high prevalence rates of STIs. By collecting detailed data on sexual behavior and host factors (such as vaginal and other microbiomes) and relating this information to incident STIs it should be possible to ascertain which factors are responsible for the emergence of differences in STI prevalence in these populations.

## References

[1] Idsoe O., Guthe T. (1967). The rise and fall of the treponematoses. I. Ecological aspects and international trends. in venereal syphilis. *British Journal of Venereal Diseases*.

[2] Aral S. O., Over M., Manhart L., Holmes K. K., Jamison D. T., Mosley W. H., Measham A. R. (2006). Sexually transmitted infections. *Disease Control Priorities in Developing Countries*.

[3] Gray R. H., Wawer M. J. (2008). Reassessing the hypothesis on STI control for HIV prevention. *The Lancet*.

[4] Corey L., Wald A., Patel R. (2004). Once-daily valacyclovir to reduce the risk of transmission of genital herpes. *The New England Journal of Medicine*.

[5] Looker K. J., Garnett G. P., Schmid G. P. (2008). An estimate of the global prevalence and incidence of herpes simplex virus type 2 infection. *Bulletin of the World Health Organization*.

[6] Kenyon C., Colebunders R., Hens N. (2013). Determinants of generalized herpes simplex virus-2 epidemics: the role of sexual partner concurrency. *International Journal of STD and AIDS*.

[7] Buvé A., Weiss H. A., Laga M. (2001). The epidemiology of gonorrhoea, chlamydial infection and syphilis in four African cities. *AIDS*.

[8] Johnson L. F., Lewis D. A. (2008). The effect of genital tract infections on HIV-1 shedding in the genital tract: a systematic review and meta-analysis. *Sexually Transmitted Diseases*.

[9] Kenyon C. R., Osbak K., Chico R. M. (2014). What underpins the decline in syphilis in Southern and Eastern Africa? An exploratory ecological analysis. *International Journal of Infectious Diseases*.

[10] Temmerman M., Fonck K., Bashir F. (1999). Declining syphilis prevalence in pregnant women in Nairobi since 1995: another success story in the STD field?. *International Journal of STD & AIDS*.

[11] Chesson H. W., Dee T. S., Aral S. O. (2003). AIDS mortality may have contributed to the decline in syphilis rates in the United States in the 1990s. *Sexually Transmitted Diseases*.

[12] Kenyon C. R., Osbak K., Buyze J., Chico R. M. (2015). The changing relationship between bacterial STIs and HIV prevalence in South Africa—an ecological study. *International Journal of STD & AIDS*.

[13] World Health Organization (2011). *Prevalence and Incidence of Selected Sexually Transmitted Infections: Chlamydia trachomatis, Neisseria gonorrhoeae, Syphilis and Trichomonas vaginalis. Methods and Results Used by WHO to Generate 2005 Estimates*.

[14] Rowley J., Toskin I., Ndowa F. (2012). Global incidence and prevalence of selected curable sexually transmitted infections, 2008.

[15] Lozano R., Naghavi M., Foreman K. (2012). Global and regional mortality from 235 causes of death for 20 age groups in 1990 and 2010: a systematic analysis for the Global Burden of Disease study 2010. *The Lancet*.

[16] Meredith S., Hawkes S., Schmid G., Broutet N. (2007). *The Global Elimination of Congenital Syphilis: Rationale and Strategy for Action*.

[17] Tramont E., Mandell G. L., Bennett J. E., Dolin R. (2010). Syphilis. *Principles and Practice of Infectious Diseases*.

[18] Cal Ham D., Lin C., Newman L., Saman Wijesooriya N., Kamb M. (2015). Improving global estimates of syphilis in pregnancy by diagnostic test type: a systematic review and meta-analysis. *International Journal of Gynecology & Obstetrics*.

[19] Looker K. J., Magaret A. S., Turner K. M. E., Vickerman P., Gottlieb S. L., Newman L. M. (2015). Correction: global estimates of prevalent and incident herpes simplex virus type 2 infections in 2012. *PLoS ONE*.

[20] Smith J. S., Robinson N. J. (2002). Age-specific prevalence of infection with herpes simplex virus types 2 and 1: a global review. *Journal of Infectious Diseases*.

[21] Weiss H. A., Thomas S. L., Munabi S. K., Hayes R. J. (2006). Male circumcision and risk of syphilis, chancroid, and genital herpes: a systematic review and meta-analysis. *Sexually Transmitted Infections*.

[22] Wamai R. G., Morris B. J., Bailis S. A. (2011). Male circumcision for HIV prevention: current evidence and implementation in sub-Saharan Africa. *Journal of the International AIDS Society*.

[23] Auvert B., Buvé A., Lagarde E. (2001). Male circumcision and HIV infection in four cities in sub-Saharan Africa. *AIDS*.

[24] Weiss H. (2008). Male circumcision: global trends and determinants of prevalence, safety, and acceptability.

[25] Kenyon C., Buyze J. (2014). Should the threshold for a generalised HIV epidemic be 1% or 5%?. *International Journal of STD and AIDS*.

[26] Osbak K. K., Rowley J. T., Kassebaum N. J., Kenyon C. R. (2016). The prevalence of syphilis from the early HIV period is correlated with peak HIV prevalence at a country level. *Sexually Transmitted Diseases*.

[27] Ward H., Rönn M. (2010). Contribution of sexually transmitted infections to the sexual transmission of HIV. *Current Opinion in HIV and AIDS*.

[28] Kenyon C. R., Osbak K., Buyze J. (2014). The prevalence of HIV by ethnic group is correlated with HSV-2 and syphilis prevalence in Kenya, South Africa, the United Kingdom, and the United States. *Interdisciplinary Perspectives on Infectious Diseases*.

[29] Grosskurth H., Todd J., Mwijarubi E. (1995). Impact of improved treatment of sexually transmitted diseases on HIV infection in rural Tanzania: randomised controlled trial. *The Lancet*.

[30] Hamilton D. T., Morris M. (2015). The racial disparities in STI in the U.S.: concurrency, STI prevalence, and heterogeneity in partner selection. *Epidemics*.

[31] Koumans E. H., Farley T. A., Gibson J. J. (2001). Characteristics of persons with syphilis in areas of persisting syphilis in the United States: sustained transmission associated with concurrent partnerships. *Sexually Transmitted Diseases*.

[32] Kenyon C., Colebunders R., Buve A., Hens N. (2013). Partner-concurrency associated with herpes simplex virus 2 infection in young South Africans. *International Journal of STD and AIDS*.

[33] Kenyon C. R., Osbak K. (2015). The prevalence of syphilis is associated with the prevalence of male point-concurrency: an ecological analysis. *World Journal of AIDS*.

[34] Kenyon C., Colebunders R. (2012). Strong association between point-concurrency and national peak HIV prevalence. *International Journal of Infectious Diseases*.

